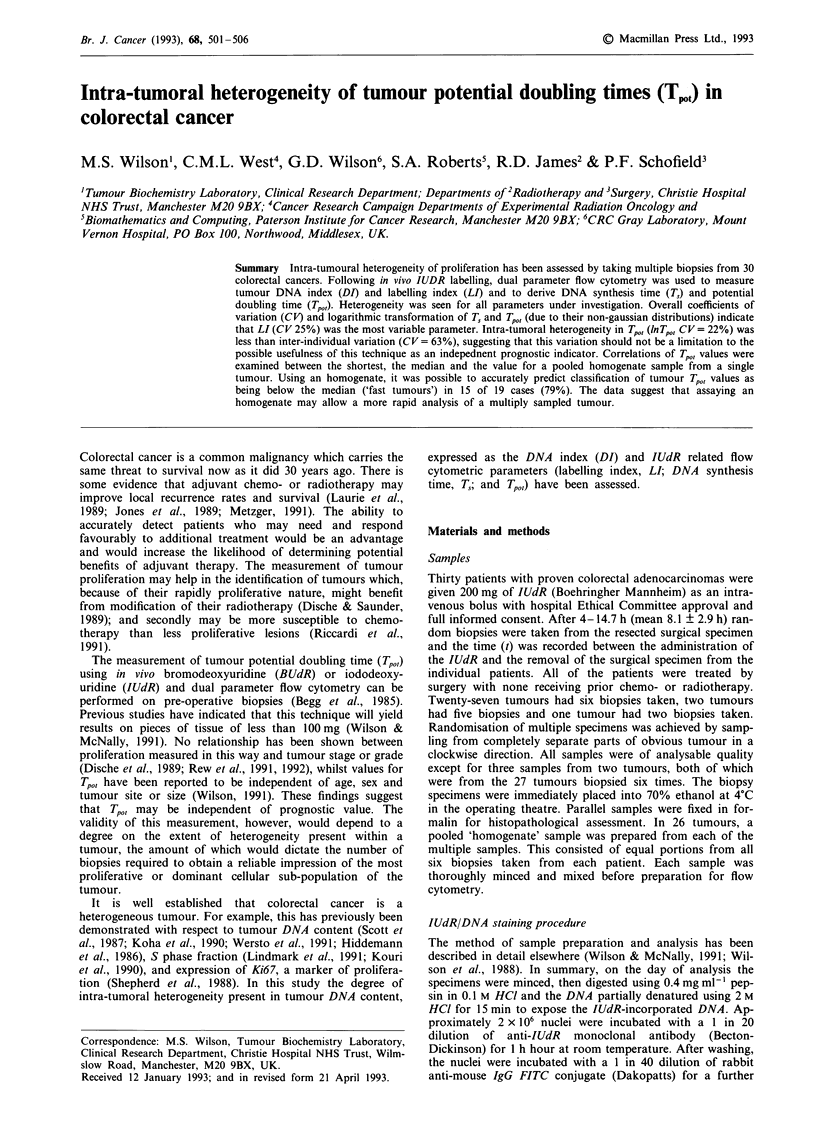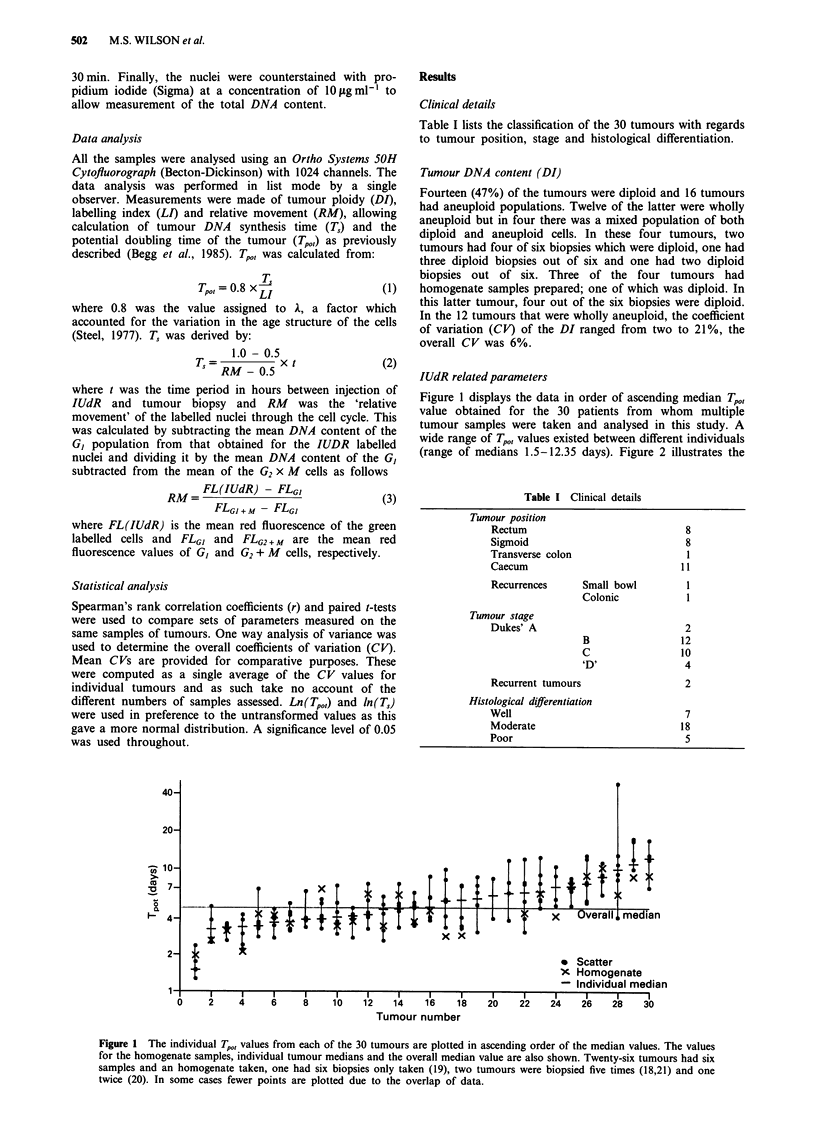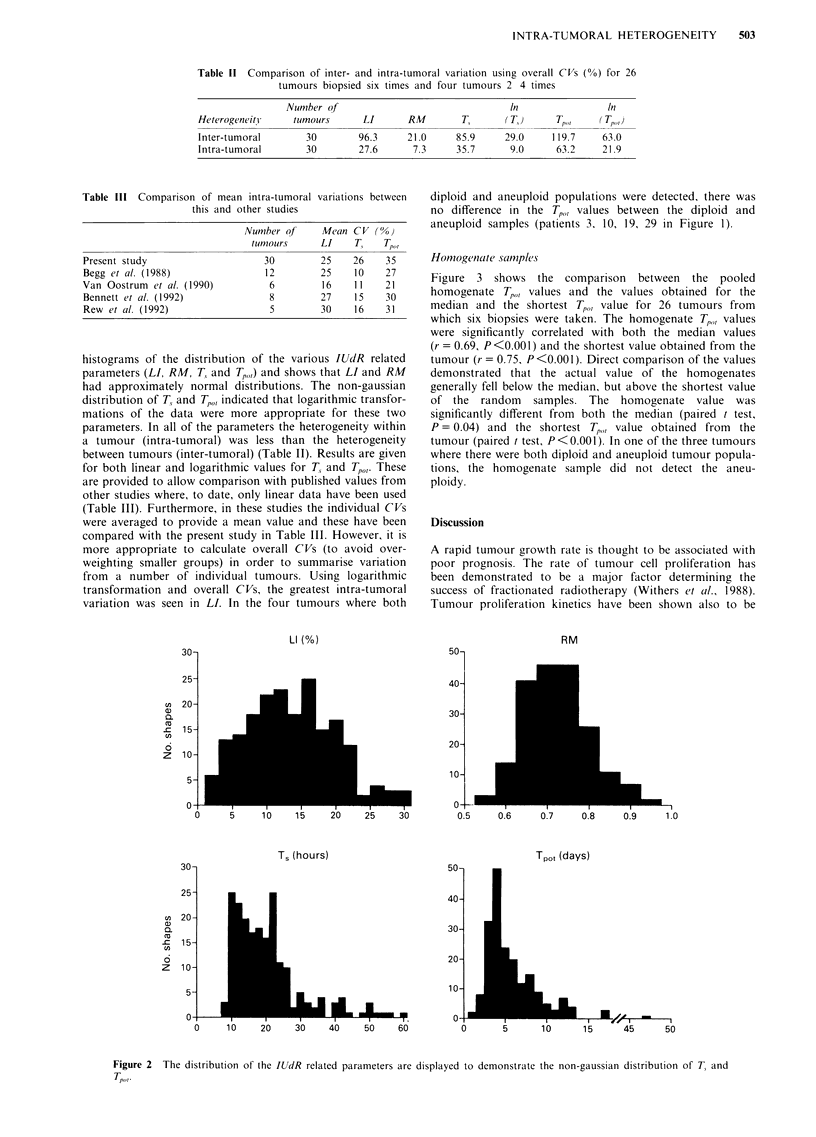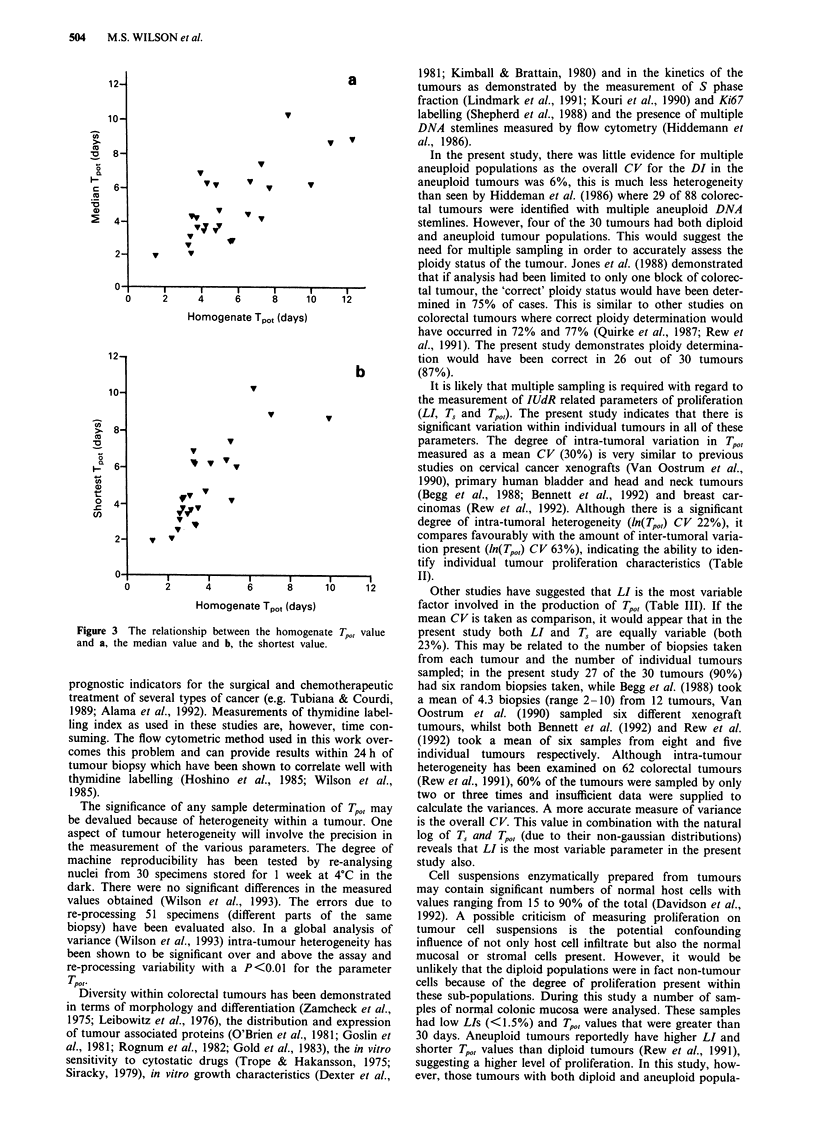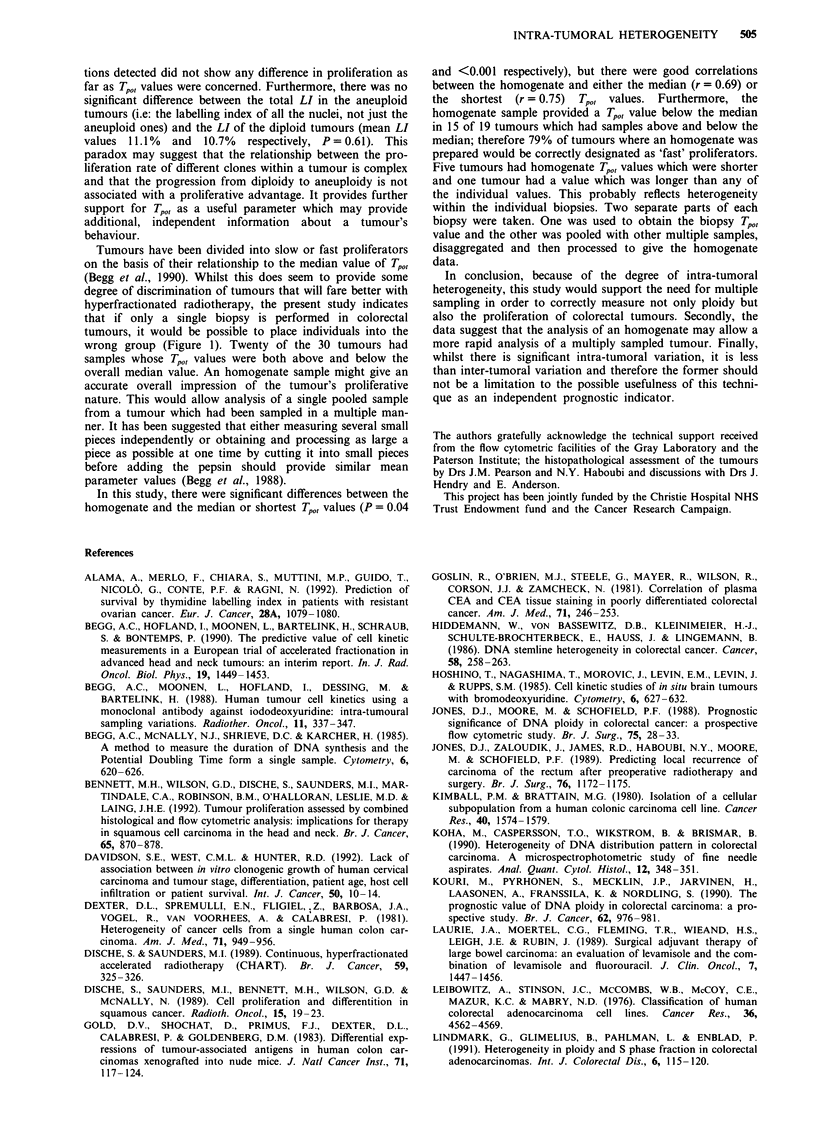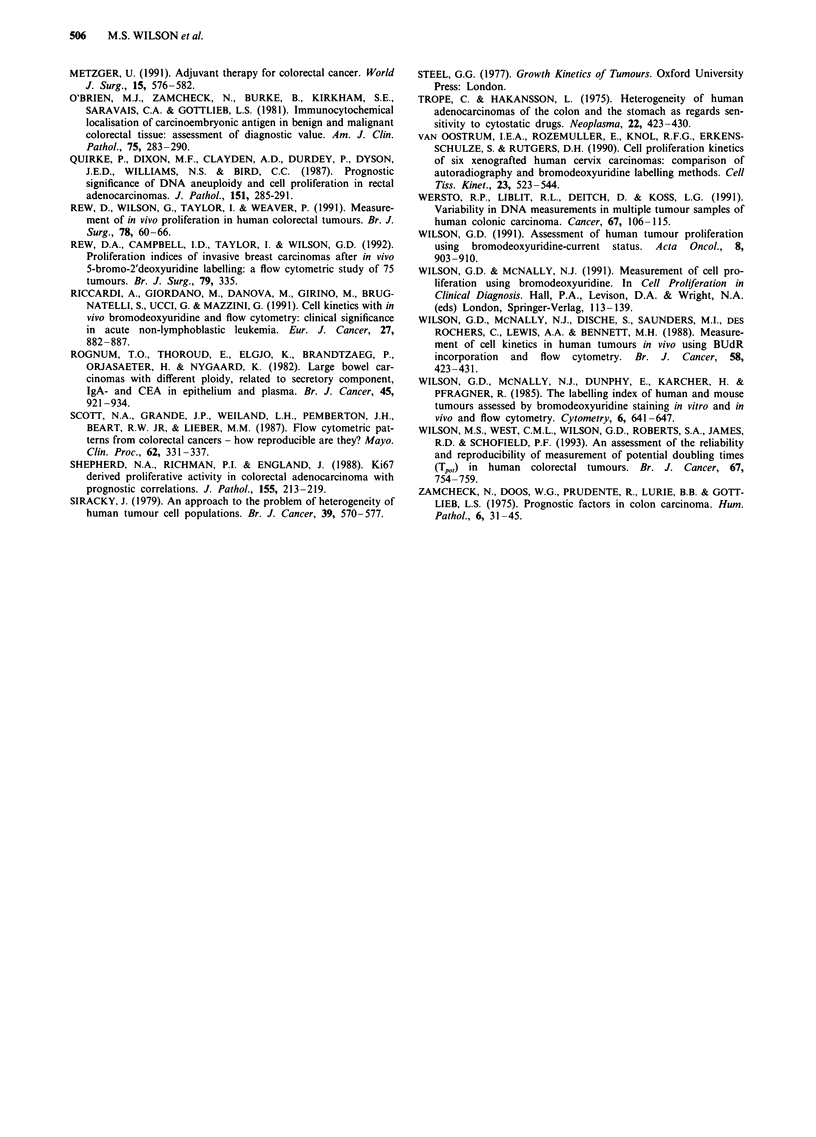# Intra-tumoral heterogeneity of tumour potential doubling times (Tpot) in colorectal cancer.

**DOI:** 10.1038/bjc.1993.376

**Published:** 1993-09

**Authors:** M. S. Wilson, C. M. West, G. D. Wilson, S. A. Roberts, R. D. James, P. F. Schofield

**Affiliations:** Clinical Research Department, Christie Hospital NHS Trust, Manchester, UK.

## Abstract

Intra-tumoural heterogeneity of proliferation has been assessed by taking multiple biopsies from 30 colorectal cancers. Following in vivo IUDR labelling, dual parameter flow cytometry was used to measure tumour DNA index (DI) and labelling index (LI) and to derive DNA synthesis time (Ts) and potential doubling time (Tpot). Heterogeneity was seen for all parameters under investigation. Overall coefficients of variation (CV) and logarithmic transformation of Ts and Tpot (due to their non-gaussian distributions) indicate that LI (CV 25%) was the most variable parameter. Intra-tumoral heterogeneity in Tpot (lnTpot CV = 22%) was less than inter-individual variation (CV = 63%), suggesting that this variation should not be a limitation to the possible usefulness of this technique as an independent prognostic indicator. Correlations of Tpot values were examined between the shortest, the median and the value for a pooled homogenate sample from a single tumour. Using an homogenate, it was possible to accurately predict classification of tumour Tpot values as being below the median ('fast tumours') in 15 of 19 cases (79%). The data suggest that assaying an homogenate may allow a more rapid analysis of a multiply sampled tumour.


Br. J. Cancer (1993), 68, 501–506
© Macmillan Press Ltd., 1993

### Intra-tumoral heterogeneity of tumour potential doubling times ($T_{pot}$) in colorectal cancer

M.S. Wilson[1], C.M.L. West[4], G.D. Wilson[6], S.A. Roberts[5], R.D. James[2] & P.F. Schofield[3]

[1]Tumour Biochemistry Laboratory, Clinical Research Department; Departments of [2]Radiotherapy and [3]Surgery, Christie Hospital NHS Trust, Manchester M20 9BX; [4]Cancer Research Campaign Departments of Experimental Radiation Oncology and [5]Biomathematics and Computing, Paterson Institute for Cancer Research, Manchester M20 9BX; [6]CRC Gray Laboratory, Mount Vernon Hospital, PO Box 100, Northwood, Middlesex, UK.

**Summary** Intra-tumoural heterogeneity of proliferation has been assessed by taking multiple biopsies from 30 colorectal cancers. Following *in vivo IUDR* labelling, dual parameter flow cytometry was used to measure tumour DNA index (*DI*) and labelling index (*LI*) and to derive DNA synthesis time ($T_s$) and potential doubling time ($T_{pot}$). Heterogeneity was seen for all parameters under investigation. Overall coefficients of variation (*CV*) and logarithmic transformation of $T_s$ and $T_{pot}$ (due to their non-gaussian distributions) indicate that *LI* (*CV* 25%) was the most variable parameter. Intra-tumoral heterogeneity in $T_{pot}$ ($lnT_{pot}$ $CV = 22\%$) was less than inter-individual variation ($CV = 63\%$), suggesting that this variation should not be a limitation to the possible usefulness of this technique as an indepednent prognostic indicator. Correlations of $T_{pot}$ values were examined between the shortest, the median and the value for a pooled homogenate sample from a single tumour. Using an homogenate, it was possible to accurately predict classification of tumour $T_{pot}$ values as being below the median ('fast tumours') in 15 of 19 cases (79%). The data suggest that assaying an homogenate may allow a more rapid analysis of a multiply sampled tumour.

Colorectal cancer is a common malignancy which carries the same threat to survival now as it did 30 years ago. There is some evidence that adjuvant chemo- or radiotherapy may improve local recurrence rates and survival (Laurie *et al.*, 1989; Jones *et al.*, 1989; Metzger, 1991). The ability to accurately detect patients who may need and respond favourably to additional treatment would be an advantage and would increase the likelihood of determining potential benefits of adjuvant therapy. The measurement of tumour proliferation may help in the identification of tumours which, because of their rapidly proliferative nature, might benefit from modification of their radiotherapy (Dische & Saunder, 1989); and secondly may be more susceptible to chemotherapy than less proliferative lesions (Riccardi *et al.*, 1991).

The measurement of tumour potential doubling time ($T_{pot}$) using *in vivo* bromodeoxyuridine (*BUdR*) or iododeoxyuridine (*IUdR*) and dual parameter flow cytometry can be performed on pre-operative biopsies (Begg *et al.*, 1985). Previous studies have indicated that this technique will yield results on pieces of tissue of less than 100 mg (Wilson & McNally, 1991). No relationship has been shown between proliferation measured in this way and tumour stage or grade (Dische *et al.*, 1989; Rew *et al.*, 1991, 1992), whilst values for $T_{pot}$ have been reported to be independent of age, sex and tumour site or size (Wilson, 1991). These findings suggest that $T_{pot}$ may be independent of prognostic value. The validity of this measurement, however, would depend to a degree on the extent of heterogeneity present within a tumour, the amount of which would dictate the number of biopsies required to obtain a reliable impression of the most proliferative or dominant cellular sub-population of the tumour.

It is well established that colorectal cancer is a heterogeneous tumour. For example, this has previously been demonstrated with respect to tumour *DNA* content (Scott *et al.*, 1987; Koha *et al.*, 1990; Wersto *et al.*, 1991; Hiddemann *et al.*, 1986), *S* phase fraction (Lindmark *et al.*, 1991; Kouri *et al.*, 1990), and expression of *Ki67*, a marker of proliferation (Shepherd *et al.*, 1988). In this study the degree of intra-tumoral heterogeneity present in tumour *DNA* content, expressed as the *DNA* index (*DI*) and *IUdR* related flow cytometric parameters (labelling index, *LI*; *DNA* synthesis time, $T_s$; and $T_{pot}$) have been assessed.

Correspondence: M.S. Wilson, Tumour Biochemistry Laboratory, Clinical Research Department, Christie Hospital NHS Trust, Wilmslow Road, Manchester, M20 9BX, UK.
Received 12 January 1993; and in revised form 21 April 1993.

### Materials and methods

### Samples

Thirty patients with proven colorectal adenocarcinomas were given 200 mg of *IUdR* (Boehringher Mannheim) as an intravenous bolus with hospital Ethical Committee approval and full informed consent. After 4–14.7 h (mean 8.1 ± 2.9 h) random biopsies were taken from the resected surgical specimen and the time (*t*) was recorded between the administration of the *IUdR* and the removal of the surgical specimen from the individual patients. All of the patients were treated by surgery with none receiving prior chemo- or radiotherapy. Twenty-seven tumours had six biopsies taken, two tumours had five biopsies and one tumour had two biopsies taken. Randomisation of multiple specimens was achieved by sampling from completely separate parts of obvious tumour in a clockwise direction. All samples were of analysable quality except for three samples from two tumours, both of which were from the 27 tumours biopsied six times. The biopsy specimens were immediately placed into 70% ethanol at 4°C in the operating theatre. Parallel samples were fixed in formalin for histopathological assessment. In 26 tumours, a pooled 'homogenate' sample was prepared from each of the multiple samples. This consisted of equal portions from all six biopsies taken from each patient. Each sample was thoroughly minced and mixed before preparation for flow cytometry.

### IUdR/DNA staining procedure

The method of sample preparation and analysis has been described in detail elsewhere (Wilson & McNally, 1991; Wilson *et al.*, 1988). In summary, on the day of analysis the specimens were minced, then digested using 0.4 mg ml$^{-1}$ pepsin in 0.1 M *HCl* and the *DNA* partially denatured using 2 M *HCl* for 15 min to expose the *IUdR*-incorporated *DNA*. Approximately $2 \times 10^6$ nuclei were incubated with a 1 in 20 dilution of anti-*IUdR* monoclonal antibody (Becton-Dickinson) for 1 h hour at room temperature. After washing, the nuclei were incubated with a 1 in 40 dilution of rabbit anti-mouse *IgG FITC* conjugate (Dakopatts) for a further

30 min. Finally, the nuclei were counterstained with propidium iodide (Sigma) at a concentration of $10\,\mu g\,ml^{-1}$ to allow measurement of the total *DNA* content.

*Data analysis*

All the samples were analysed using an *Ortho Systems 50H Cytofluorograph* (Becton-Dickinson) with 1024 channels. The data analysis was performed in list mode by a single observer. Measurements were made of tumour ploidy (*DI*), labelling index (*LI*) and relative movement (*RM*), allowing calculation of tumour *DNA* synthesis time ($T_s$) and the potential doubling time of the tumour ($T_{pot}$) as previously described (Begg *et al.*, 1985). $T_{pot}$ was calculated from:

$$T_{pot} = 0.8 \times \frac{T_s}{LI} \quad (1)$$

where 0.8 was the value assigned to $\lambda$, a factor which accounted for the variation in the age structure of the cells (Steel, 1977). $T_s$ was derived by:

$$T_s = \frac{1.0 - 0.5}{RM - 0.5} \times t \quad (2)$$

where $t$ was the time period in hours between injection of *IUdR* and tumour biopsy and *RM* was the 'relative movement' of the labelled nuclei through the cell cycle. This was calculated by subtracting the mean *DNA* content of the $G_1$ population from that obtained for the *IUDR* labelled nuclei and dividing it by the mean *DNA* content of the $G_1$ subtracted from the mean of the $G_2 \times M$ cells as follows

$$RM = \frac{FL(IUdR) - FL_{G1}}{FL_{G1+M} - FL_{G1}} \quad (3)$$

where $FL(IUdR)$ is the mean red fluorescence of the green labelled cells and $FL_{G1}$ and $FL_{G2+M}$ are the mean red fluorescence values of $G_1$ and $G_2 + M$ cells, respectively.

*Statistical analysis*

Spearman's rank correlation coefficients ($r$) and paired $t$-tests were used to compare sets of parameters measured on the same samples of tumours. One way analysis of variance was used to determine the overall coefficients of variation (*CV*). Mean *CV*s are provided for comparative purposes. These were computed as a single average of the *CV* values for individual tumours and as such take no account of the different numbers of samples assessed. $Ln(T_{pot})$ and $ln(T_s)$ were used in preference to the untransformed values as this gave a more normal distribution. A significance level of 0.05 was used throughout.

### Results

*Clinical details*

Table I lists the classification of the 30 tumours with regards to tumour position, stage and histological differentiation.

*Tumour DNA content (DI)*

Fourteen (47%) of the tumours were diploid and 16 tumours had aneuploid populations. Twelve of the latter were wholly aneuploid but in four there was a mixed population of both diploid and aneuploid cells. In these four tumours, two tumours had four of six biopsies which were diploid, one had three diploid biopsies out of six and one had two diploid biopsies out of six. Three of the four tumours had homogenate samples prepared; one of which was diploid. In this latter tumour, four out of the six biopsies were diploid. In the 12 tumours that were wholly aneuploid, the coefficient of variation (*CV*) of the *DI* ranged from two to 21%, the overall *CV* was 6%.

*IUdR related parameters*

Figure 1 displays the data in order of ascending median $T_{pot}$ value obtained for the 30 patients from whom multiple tumour samples were taken and analysed in this study. A wide range of $T_{pot}$ values existed between different individuals (range of medians 1.5–12.35 days). Figure 2 illustrates the

**Table I** Clinical details

| | | |
|---|---|---|
| *Tumour position* | | |
| Rectum | | 8 |
| Sigmoid | | 8 |
| Transverse colon | | 1 |
| Caecum | | 11 |
| Recurrences | Small bowl | 1 |
| | Colonic | 1 |
| *Tumour stage* | | |
| Dukes' A | | 2 |
| | B | 12 |
| | C | 10 |
| | 'D' | 4 |
| Recurrent tumours | | 2 |
| *Histological differentiation* | | |
| Well | | 7 |
| Moderate | | 18 |
| Poor | | 5 |

**Figure 1** The individual $T_{pot}$ values from each of the 30 tumours are plotted in ascending order of the median values. The values for the homogenate samples, individual tumour medians and the overall median value are also shown. Twenty-six tumours had six samples and an homogenate taken, one had six biopsies only taken (19), two tumours were biopsied five times (18,21) and one twice (20). In some cases fewer points are plotted due to the overlap of data.

**Table II** Comparison of inter- and intra-tumoral variation using overall *CVs* (%) for 26 tumours biopsied six times and four tumours 2 4 times

| *Heterogeneity* | *Number of tumours* | *LI* | *RM* | $T_s$ | *ln* $(T_s)$ | $T_{pot}$ | *ln* $(T_{pot})$ |
|---|---|---|---|---|---|---|---|
| Inter-tumoral | 30 | 96.3 | 21.0 | 85.9 | 29.0 | 119.7 | 63.0 |
| Intra-tumoral | 30 | 27.6 | 7.3 | 35.7 | 9.0 | 63.2 | 21.9 |

**Table III** Comparison of mean intra-tumoral variations between this and other studies

| | *Number of tumours* | *Mean CV (%)* *LI* | $T_s$ | $T_{pot}$ |
|---|---|---|---|---|
| Present study | 30 | 25 | 26 | 35 |
| Begg *et al.* (1988) | 12 | 25 | 10 | 27 |
| Van Oostrum *et al.* (1990) | 6 | 16 | 11 | 21 |
| Bennett *et al.* (1992) | 8 | 27 | 15 | 30 |
| Rew *et al.* (1992) | 5 | 30 | 16 | 31 |

histograms of the distribution of the various *IUdR* related parameters (*LI*, *RM*, $T_s$ and $T_{pot}$) and shows that *LI* and *RM* had approximately normal distributions. The non-gaussian distribution of $T_s$ and $T_{pot}$ indicated that logarithmic transformations of the data were more appropriate for these two parameters. In all of the parameters the heterogeneity within a tumour (intra-tumoral) was less than the heterogeneity between tumours (inter-tumoral) (Table II). Results are given for both linear and logarithmic values for $T_s$ and $T_{pot}$. These are provided to allow comparison with published values from other studies where, to date, only linear data have been used (Table III). Furthermore, in these studies the individual *CVs* were averaged to provide a mean value and these have been compared with the present study in Table III. However, it is more appropriate to calculate overall *CVs* (to avoid overweighting smaller groups) in order to summarise variation from a number of individual tumours. Using logarithmic transformation and overall *CVs*, the greatest intra-tumoral variation was seen in *LI*. In the four tumours where both diploid and aneuploid populations were detected, there was no difference in the $T_{pot}$ values between the diploid and aneuploid samples (patients 3, 10, 19, 29 in Figure 1).

*Homogenate samples*

Figure 3 shows the comparison between the pooled homogenate $T_{pot}$ values and the values obtained for the median and the shortest $T_{pot}$ value for 26 tumours from which six biopsies were taken. The homogenate $T_{pot}$ values were significantly correlated with both the median values ($r = 0.69$, $P<0.001$) and the shortest value obtained from the tumour ($r = 0.75$, $P<0.001$). Direct comparison of the values demonstrated that the actual value of the homogenates generally fell below the median, but above the shortest value of the random samples. The homogenate value was significantly different from both the median (paired *t* test, $P = 0.04$) and the shortest $T_{pot}$ value obtained from the tumour (paired *t* test, $P<0.001$). In one of the three tumours where there were both diploid and aneuploid tumour populations, the homogenate sample did not detect the aneuploidy.

### Discussion

A rapid tumour growth rate is thought to be associated with poor prognosis. The rate of tumour cell proliferation has been demonstrated to be a major factor determining the success of fractionated radiotherapy (Withers *et al.*, 1988). Tumour proliferation kinetics have been shown also to be

**Figure 2** The distribution of the *IUdR* related parameters are displayed to demonstrate the non-gaussian distribution of $T_s$ and $T_{pot}$.

**Figure 3** The relationship between the homogenate $T_{pot}$ value and **a**, the median value and **b**, the shortest value.

prognostic indicators for the surgical and chemotherapeutic treatment of several types of cancer (e.g. Tubiana & Courdi, 1989; Alama *et al.*, 1992). Measurements of thymidine labelling index as used in these studies are, however, time consuming. The flow cytometric method used in this work overcomes this problem and can provide results within 24 h of tumour biopsy which have been shown to correlate well with thymidine labelling (Hoshino *et al.*, 1985; Wilson *et al.*, 1985).

The significance of any sample determination of $T_{pot}$ may be devalued because of heterogeneity within a tumour. One aspect of tumour heterogeneity will involve the precision in the measurement of the various parameters. The degree of machine reproducibility has been tested by re-analysing nuclei from 30 specimens stored for 1 week at 4°C in the dark. There were no significant differences in the measured values obtained (Wilson *et al.*, 1993). The errors due to re-processing 51 specimens (different parts of the same biopsy) have been evaluated also. In a global analysis of variance (Wilson *et al.*, 1993) intra-tumour heterogeneity has been shown to be significant over and above the assay and re-processing variability with a $P<0.01$ for the parameter $T_{pot}$.

Diversity within colorectal tumours has been demonstrated in terms of morphology and differentiation (Zamcheck *et al.*, 1975; Leibowitz *et al.*, 1976), the distribution and expression of tumour associated proteins (O'Brien *et al.*, 1981; Goslin *et al.*, 1981; Rognum *et al.*, 1982; Gold *et al.*, 1983), the *in vitro* sensitivity to cytostatic drugs (Trope & Hakansson, 1975; Siracky, 1979), *in vitro* growth characteristics (Dexter *et al.*, 1981; Kimball & Brattain, 1980) and in the kinetics of the tumours as demonstrated by the measurement of *S* phase fraction (Lindmark *et al.*, 1991; Kouri *et al.*, 1990) and *Ki67* labelling (Shepherd *et al.*, 1988) and the presence of multiple *DNA* stemlines measured by flow cytometry (Hiddemann *et al.*, 1986).

In the present study, there was little evidence for multiple aneuploid populations as the overall *CV* for the *DI* in the aneuploid tumours was 6%, this is much less heterogeneity than seen by Hiddeman *et al.* (1986) where 29 of 88 colorectal tumours were identified with multiple aneuploid *DNA* stemlines. However, four of the 30 tumours had both diploid and aneuploid tumour populations. This would suggest the need for multiple sampling in order to accurately assess the ploidy status of the tumour. Jones *et al.* (1988) demonstrated that if analysis had been limited to only one block of colorectal tumour, the 'correct' ploidy status would have been determined in 75% of cases. This is similar to other studies on colorectal tumours where correct ploidy determination would have occurred in 72% and 77% (Quirke *et al.*, 1987; Rew *et al.*, 1991). The present study demonstrates ploidy determination would have been correct in 26 out of 30 tumours (87%).

It is likely that multiple sampling is required with regard to the measurement of *IUdR* related parameters of proliferation (*LI*, $T_s$ and $T_{pot}$). The present study indicates that there is significant variation within individual tumours in all of these parameters. The degree of intra-tumoral variation in $T_{pot}$ measured as a mean *CV* (30%) is very similar to previous studies on cervical cancer xenografts (Van Oostrum *et al.*, 1990), primary human bladder and head and neck tumours (Begg *et al.*, 1988; Bennett *et al.*, 1992) and breast carcinomas (Rew *et al.*, 1992). Although there is a significant degree of intra-tumoral heterogeneity ($ln(T_{pot})$ *CV* 22%), it compares favourably with the amount of inter-tumoral variation present ($ln(T_{pot})$ *CV* 63%), indicating the ability to identify individual tumour proliferation characteristics (Table II).

Other studies have suggested that *LI* is the most variable factor involved in the production of $T_{pot}$ (Table III). If the mean *CV* is taken as comparison, it would appear that in the present study both *LI* and $T_s$ are equally variable (both 23%). This may be related to the number of biopsies taken from each tumour and the number of individual tumours sampled; in the present study 27 of the 30 tumours (90%) had six random biopsies taken, while Begg *et al.* (1988) took a mean of 4.3 biopsies (range 2–10) from 12 tumours, Van Oostrum *et al.* (1990) sampled six different xenograft tumours, whilst both Bennett *et al.* (1992) and Rew *et al.* (1992) took a mean of six samples from eight and five individual tumours respectively. Although intra-tumour heterogeneity has been examined on 62 colorectal tumours (Rew *et al.*, 1991), 60% of the tumours were sampled by only two or three times and insufficient data were supplied to calculate the variances. A more accurate measure of variance is the overall *CV*. This value in combination with the natural log of $T_s$ *and* $T_{pot}$ (due to their non-gaussian distributions) reveals that *LI* is the most variable parameter in the present study also.

Cell suspensions enzymatically prepared from tumours may contain significant numbers of normal host cells with values ranging from 15 to 90% of the total (Davidson *et al.*, 1992). A possible criticism of measuring proliferation on tumour cell suspensions is the potential confounding influence of not only host cell infiltrate but also the normal mucosal or stromal cells present. However, it would be unlikely that the diploid populations were in fact non-tumour cells because of the degree of proliferation present within these sub-populations. During this study a number of samples of normal colonic mucosa were analysed. These samples had low *LI*s (<1.5%) and $T_{pot}$ values that were greater than 30 days. Aneuploid tumours reportedly have higher *LI* and shorter $T_{pot}$ values than diploid tumours (Rew *et al.*, 1991), suggesting a higher level of proliferation. In this study, however, those tumours with both diploid and aneuploid populations detected did not show any difference in proliferation as far as $T_{pot}$ values were concerned. Furthermore, there was no significant difference between the total *LI* in the aneuploid tumours (i.e: the labelling index of all the nuclei, not just the aneuploid ones) and the *LI* of the diploid tumours (mean *LI* values 11.1% and 10.7% respectively, $P = 0.61$). This paradox may suggest that the relationship between the proliferation rate of different clones within a tumour is complex and that the progression from diploidy to aneuploidy is not associated with a proliferative advantage. It provides further support for $T_{pot}$ as a useful parameter which may provide additional, independent information about a tumour's behaviour.

Tumours have been divided into slow or fast proliferators on the basis of their relationship to the median value of $T_{pot}$ (Begg *et al.*, 1990). Whilst this does seem to provide some degree of discrimination of tumours that will fare better with hyperfractionated radiotherapy, the present study indicates that if only a single biopsy is performed in colorectal tumours, it would be possible to place individuals into the wrong group (Figure 1). Twenty of the 30 tumours had samples whose $T_{pot}$ values were both above and below the overall median value. An homogenate sample might give an accurate overall impression of the tumour's proliferative nature. This would allow analysis of a single pooled sample from a tumour which had been sampled in a multiple manner. It has been suggested that either measuring several small pieces independently or obtaining and processing as large a piece as possible at one time by cutting it into small pieces before adding the pepsin should provide similar mean parameter values (Begg *et al.*, 1988).

In this study, there were significant differences between the homogenate and the median or shortest $T_{pot}$ values ($P = 0.04$ and $<0.001$ respectively), but there were good correlations between the homogenate and either the median ($r = 0.69$) or the shortest ($r = 0.75$) $T_{pot}$ values. Furthermore, the homogenate sample provided a $T_{pot}$ value below the median in 15 of 19 tumours which had samples above and below the median; therefore 79% of tumours where an homogenate was prepared would be correctly designated as 'fast' proliferators. Five tumours had homogenate $T_{pot}$ values which were shorter and one tumour had a value which was longer than any of the individual values. This probably reflects heterogeneity within the individual biopsies. Two separate parts of each biopsy were taken. One was used to obtain the biopsy $T_{pot}$ value and the other was pooled with other multiple samples, disaggregated and then processed to give the homogenate data.

In conclusion, because of the degree of intra-tumoral heterogeneity, this study would support the need for multiple sampling in order to correctly measure not only ploidy but also the proliferation of colorectal tumours. Secondly, the data suggest that the analysis of an homogenate may allow a more rapid analysis of a multiply sampled tumour. Finally, whilst there is significant intra-tumoral variation, it is less than inter-tumoral variation and therefore the former should not be a limitation to the possible usefulness of this technique as an independent prognostic indicator.

The authors gratefully acknowledge the technical support received from the flow cytometric facilities of the Gray Laboratory and the Paterson Institute; the histopathological assessment of the tumours by Drs J.M. Pearson and N.Y. Haboubi and discussions with Drs J. Hendry and E. Anderson.

This project has been jointly funded by the Christie Hospital NHS Trust Endowment fund and the Cancer Research Campaign.

### References

ALAMA, A., MERLO, F., CHIARA, S., MUTTINI, M.P., GUIDO, T., NICOLÒ, G., CONTE, P.F. & RAGNI, N. (1992). Prediction of survival by thymidine labelling index in patients with resistant ovarian cancer. *Eur. J. Cancer*, **28A**, 1079–1080.

BEGG, A.C., HOFLAND, I., MOONEN, L., BARTELINK, H., SCHRAUB, S. & BONTEMPS, P. (1990). The predictive value of cell kinetic measurements in a European trial of accelerated fractionation in advanced head and neck tumours: an interim report. *In. J. Rad. Oncol. Biol. Phys.*, **19**, 1449–1453.

BEGG, A.C., MOONEN, L., HOFLAND, I., DESSING, M. & BARTELINK, H. (1988). Human tumour cell kinetics using a monoclonal antibody against iododeoxyuridine: intra-tumoural sampling variations. *Radiother. Oncol.*, **11**, 337–347.

BEGG, A.C., MCNALLY, N.J., SHRIEVE, D.C. & KARCHER, H. (1985). A method to measure the duration of DNA synthesis and the Potential Doubling Time form a single sample. *Cytometry*, **6**, 620–626.

BENNETT, M.H., WILSON, G.D., DISCHE, S., SAUNDERS, M.I., MARTINDALE, C.A., ROBINSON, B.M., O'HALLORAN, LESLIE, M.D. & LAING, J.H.E. (1992). Tumour proliferation assessed by combined histological and flow cytometric analysis: implications for therapy in squamous cell carcinoma in the head and neck. *Br. J. Cancer*, **65**, 870–878.

DAVIDSON, S.E., WEST, C.M.L. & HUNTER, R.D. (1992). Lack of association between *in vitro* clonogenic growth of human cervical carcinoma and tumour stage, differentiation, patient age, host cell infiltration or patient survival. *Int. J. Cancer*, **50**, 10–14.

DEXTER, D.L., SPREMULLI, E.N., FLIGIEL, Z., BARBOSA, J.A., VOGEL, R., VAN VOORHEES, A. & CALABRESI, P. (1981). Heterogeneity of cancer cells from a single human colon carcinoma. *Am. J. Med.*, **71**, 949–956.

DISCHE, S. & SAUNDERS, M.I. (1989). Continuous, hyperfractionated accelerated radiotherapy (CHART). *Br. J. Cancer*, **59**, 325–326.

DISCHE, S., SAUNDERS, M.I., BENNETT, M.H., WILSON, G.D. & MCNALLY, N. (1989). Cell proliferation and differentition in squamous cancer. *Radioth. Oncol.*, **15**, 19–23.

GOLD, D.V., SHOCHAT, D., PRIMUS, F.J., DEXTER, D.L., CALABRESI, P. & GOLDENBERG, D.M. (1983). Differential expressions of tumour-associated antigens in human colon carcinomas xenografted into nude mice. *J. Natl Cancer Inst.*, **71**, 117–124.

GOSLIN, R., O'BRIEN, M.J., STEELE, G., MAYER, R., WILSON, R., CORSON, J.J. & ZAMCHECK, N. (1981). Correlation of plasma CEA and CEA tissue staining in poorly differentiated colorectal cancer. *Am. J. Med.*, **71**, 246–253.

HIDDEMANN, W., VON BASSEWITZ, D.B., KLEINIMEIER, H.-J., SCHULTE-BROCHTERBECK, E., HAUSS, J. & LINGEMANN, B. (1986). DNA stemline heterogeneity in colorectal cancer. *Cancer*, **58**, 258–263.

HOSHINO, T., NAGASHIMA, T., MOROVIC, J., LEVIN, E.M., LEVIN, J. & RUPPS, S.M. (1985). Cell kinetic studies of *in situ* brain tumours with bromodeoxyuridine. *Cytometry*, **6**, 627–632.

JONES, D.J., MOORE, M. & SCHOFIELD, P.F. (1988). Prognostic significance of DNA ploidy in colorectal cancer: a prospective flow cytometric study. *Br. J. Surg.*, **75**, 28–33.

JONES, D.J., ZALOUDIK, J., JAMES, R.D., HABOUBI, N.Y., MOORE, M. & SCHOFIELD, P.F. (1989). Predicting local recurrence of carcinoma of the rectum after preoperative radiotherapy and surgery. *Br. J. Surg.*, **76**, 1172–1175.

KIMBALL, P.M. & BRATTAIN, M.G. (1980). Isolation of a cellular subpopulation from a human colonic carcinoma cell line. *Cancer Res.*, **40**, 1574–1579.

KOHA, M., CASPERSSON, T.O., WIKSTROM, B. & BRISMAR, B. (1990). Heterogeneity of DNA distribution pattern in colorectal carcinoma. A microspectrophotometric study of fine needle aspirates. *Anal. Quant. Cytol. Histol.*, **12**, 348–351.

KOURI, M., PYRHONEN, S., MECKLIN, J.P., JARVINEN, H., LAASONEN, A., FRANSSILA, K. & NORDLING, S. (1990). The prognostic value of DNA ploidy in colorectal carcinoma: a prospective study. *Br. J. Cancer*, **62**, 976–981.

LAURIE, J.A., MOERTEL, C.G., FLEMING, T.R., WIEAND, H.S., LEIGH, J.E. & RUBIN, J. (1989). Surgical adjuvant therapy of large bowel carcinoma: an evaluation of levamisole and the combination of levamisole and fluorouracil. *J. Clin. Oncol.*, **7**, 1447–1456.

LEIBOWITZ, A., STINSON, J.C., MCCOMBS, W.B., MCCOY, C.E., MAZUR, K.C. & MABRY, N.D. (1976). Classification of human colorectal adenocarcinoma cell lines. *Cancer Res.*, **36**, 4562–4569.

LINDMARK, G., GLIMELIUS, B., PAHLMAN, L. & ENBLAD, P. (1991). Heterogeneity in ploidy and S phase fraction in colorectal adenocarcinomas. *Int. J. Colorectal Dis.*, **6**, 115–120.

METZGER, U. (1991). Adjuvant therapy for colorectal cancer. *World J. Surg.*, **15**, 576–582.

O'BRIEN, M.J., ZAMCHECK, N., BURKE, B., KIRKHAM, S.E., SARAVAIS, C.A. & GOTTLIEB, L.S. (1981). Immunocytochemical localisation of carcinoembryonic antigen in benign and malignant colorectal tissue: assessment of diagnostic value. *Am. J. Clin. Pathol.*, **75**, 283–290.

QUIRKE, P., DIXON, M.F., CLAYDEN, A.D., DURDEY, P., DYSON, J.E.D., WILLIAMS, N.S. & BIRD, C.C. (1987). Prognostic significance of DNA aneuploidy and cell proliferation in rectal adenocarcinomas. *J. Pathol.*, **151**, 285-291.

REW, D., WILSON, G., TAYLOR, I. & WEAVER, P. (1991). Measurement of *in vivo* proliferation in human colorectal tumours. *Br. J. Surg.*, **78**, 60–66.

REW, D.A., CAMPBELL, I.D., TAYLOR, I. & WILSON, G.D. (1992). Proliferation indices of invasive breast carcinomas after *in vivo* 5-bromo-2'deoxyuridine labelling: a flow cytometric study of 75 tumours. *Br. J. Surg.*, **79**, 335.

RICCARDI, A., GIORDANO, M., DANOVA, M., GIRINO, M., BRUGNATELLI, S., UCCI, G. & MAZZINI, G. (1991). Cell kinetics with *in vivo* bromodeoxyuridine and flow cytometry: clinical significance in acute non-lymphoblastic leukemia. *Eur. J. Cancer*, **27**, 882–887.

ROGNUM, T.O., THOROUD, E., ELGJO, K., BRANDTZAEG, P., ORJASAETER, H. & NYGAARD, K. (1982). Large bowel carcinomas with different ploidy, related to secretory component, IgA- and CEA in epithelium and plasma. *Br. J. Cancer*, **45**, 921–934.

SCOTT, N.A., GRANDE, J.P., WEILAND, L.H., PEMBERTON, J.H., BEART, R.W. JR, & LIEBER, M.M. (1987). Flow cytometric patterns from colorectal cancers – how reproducible are they? *Mayo. Clin. Proc.*, **62**, 331–337.

SHEPHERD, N.A., RICHMAN, P.I. & ENGLAND, J. (1988). Ki67 derived proliferative activity in colorectal adenocarcinoma with prognostic correlations. *J. Pathol.*, **155**, 213–219.

SIRACKY, J. (1979). An approach to the problem of heterogeneity of human tumour cell populations. *Br. J. Cancer*, **39**, 570–577.

STEEL, G.G. (1977). *Growth Kinetics of Tumours*. Oxford University Press: London.

TROPE, C. & HAKANSSON, L. (1975). Heterogeneity of human adenocarcinomas of the colon and the stomach as regards sensitivity to cytostatic drugs. *Neoplasma*, **22**, 423–430.

VAN OOSTRUM, I.E.A., ROZEMULLER, E., KNOL, R.F.G., ERKENS-SCHULZE, S. & RUTGERS, D.H. (1990). Cell proliferation kinetics of six xenografted human cervix carcinomas: comparison of autoradiography and bromodeoxyuridine labelling methods. *Cell Tiss. Kinet.*, **23**, 523–544.

WERSTO, R.P., LIBLIT, R.L., DEITCH, D. & KOSS, L.G. (1991). Variability in DNA measurements in multiple tumour samples of human colonic carcinoma. *Cancer*, **67**, 106–115.

WILSON, G.D. (1991). Assessment of human tumour proliferation using bromodeoxyuridine-current status. *Acta Oncol.*, **8**, 903–910.

WILSON, G.D. & MCNALLY, N.J. (1991). Measurement of cell proliferation using bromodeoxyuridine. In *Cell Proliferation in Clinical Diagnosis*. Hall, P.A., Levison, D.A. & Wright, N.A. (eds) London, Springer-Verlag, 113–139.

WILSON, G.D., MCNALLY, N.J., DISCHE, S., SAUNDERS, M.I., DES ROCHERS, C., LEWIS, A.A. & BENNETT, M.H. (1988). Measurement of cell kinetics in human tumours *in vivo* using BUdR incorporation and flow cytometry. *Br. J. Cancer*, **58**, 423–431.

WILSON, G.D., MCNALLY, N.J., DUNPHY, E., KARCHER, H. & PFRAGNER, R. (1985). The labelling index of human and mouse tumours assessed by bromodeoxyuridine staining *in vitro* and *in vivo* and flow cytometry. *Cytometry*, **6**, 641–647.

WILSON, M.S., WEST, C.M.L., WILSON, G.D., ROBERTS, S.A., JAMES, R.D. & SCHOFIELD, P.F. (1993). An assessment of the reliability and reproducibility of measurement of potential doubling times ($T_{pot}$) in human colorectal tumours. *Br. J. Cancer*, **67**, 754–759.

ZAMCHECK, N., DOOS, W.G., PRUDENTE, R., LURIE, B.B. & GOTTLIEB, L.S. (1975). Prognostic factors in colon carcinoma. *Hum. Pathol.*, **6**, 31–45.